# Targeting Phospholipid Metabolism in Cancer

**DOI:** 10.3389/fonc.2016.00266

**Published:** 2016-12-27

**Authors:** Menglin Cheng, Zaver M. Bhujwalla, Kristine Glunde

**Affiliations:** ^1^Division of Cancer Imaging Research, Russell H. Morgan Department of Radiology and Radiological Science, Johns Hopkins University School of Medicine, Baltimore, MD, USA; ^2^Sidney Kimmel Comprehensive Cancer Center, Johns Hopkins University School of Medicine, Baltimore, MD, USA

**Keywords:** cancer, target, phospholipid, metabolism, choline, ethanolamine

## Abstract

All cancers tested so far display abnormal choline and ethanolamine phospholipid metabolism, which has been detected with numerous magnetic resonance spectroscopy (MRS) approaches in cells, animal models of cancer, as well as the tumors of cancer patients. Since the discovery of this metabolic hallmark of cancer, many studies have been performed to elucidate the molecular origins of deregulated choline metabolism, to identify targets for cancer treatment, and to develop MRS approaches that detect choline and ethanolamine compounds for clinical use in diagnosis and treatment monitoring. Several enzymes in choline, and recently also ethanolamine, phospholipid metabolism have been identified, and their evaluation has shown that they are involved in carcinogenesis and tumor progression. Several already established enzymes as well as a number of emerging enzymes in phospholipid metabolism can be used as treatment targets for anticancer therapy, either alone or in combination with other chemotherapeutic approaches. This review summarizes the current knowledge of established and relatively novel targets in phospholipid metabolism of cancer, covering choline kinase α, phosphatidylcholine-specific phospholipase D1, phosphatidylcholine-specific phospholipase C, sphingomyelinases, choline transporters, glycerophosphodiesterases, phosphatidylethanolamine N-methyltransferase, and ethanolamine kinase. These enzymes are discussed in terms of their roles in oncogenic transformation, tumor progression, and crucial cancer cell properties such as fast proliferation, migration, and invasion. Their potential as treatment targets are evaluated based on the current literature.

## Introduction

Phospholipids, which form the bilayer structures of all cellular membranes, are an essential component of all cells. The phospholipid content was shown to increase with cell transformation and tumor progression (1–4). For example, lipid analyses of samples from breast cancer tissue displayed an increase in phospholipid content as compared to non-cancerous adjacent healthy breast tissue (5). Concentrations of the two major phospholipid components phosphatidylcholine (PtdCho) and phosphatidylethanolamine (PtdEtn) increased with increasing breast cancer tumor grade (6), indicating that the phospholipid synthesis rate increases with oncogenesis and tumor progression as compared to normal tissue.

**Figure 1:**
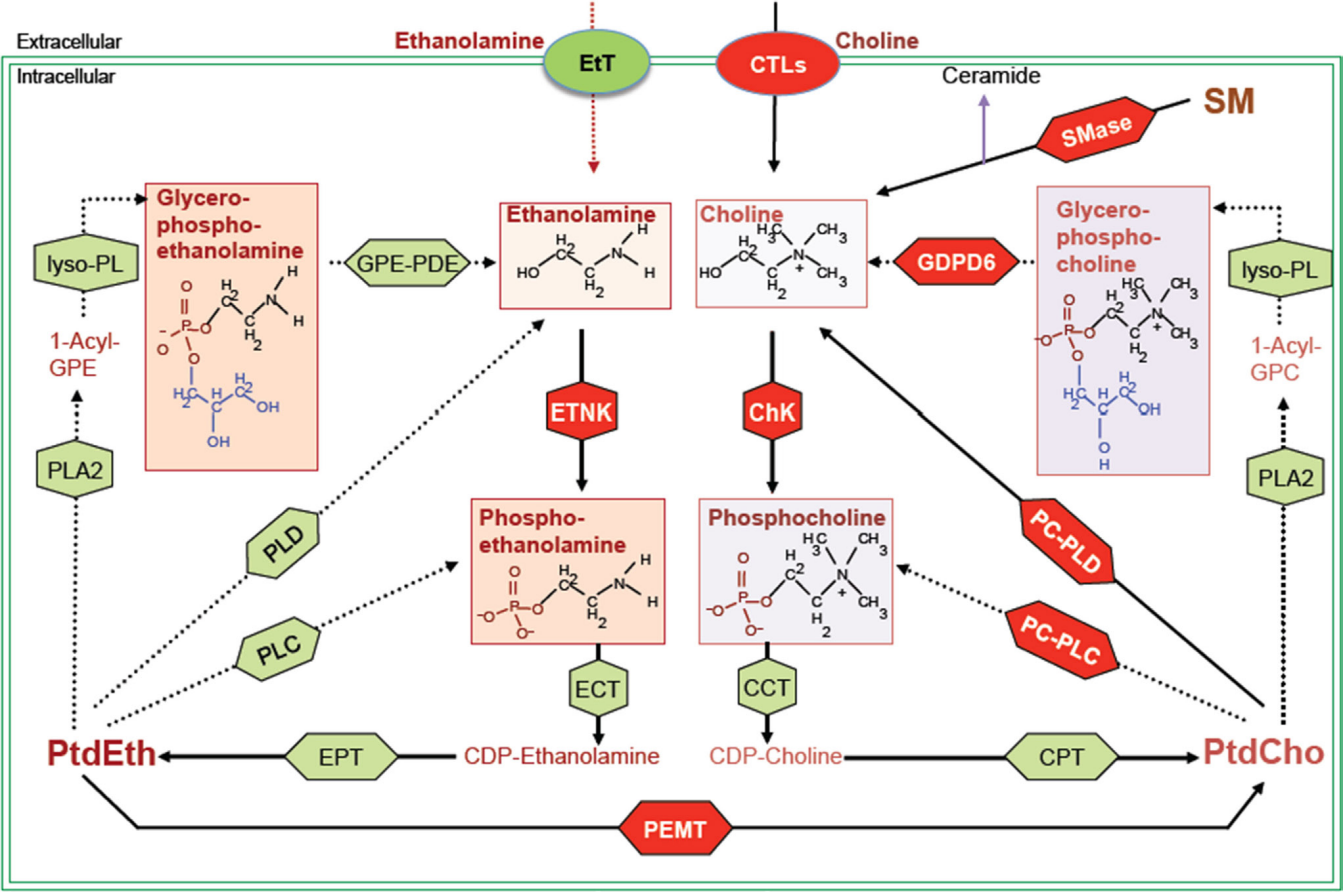
**Biochemical network of ethanolamine and choline phospholipid metabolism showing crosstalk between these two metabolic cycles**. MR detectable metabolites are drawn in boxes. Red shapes show enzymes discussed in this review, and green shapes show other enzymes responsible for the indicated reactions, but not discussed in this review. Metabolite abbreviations: 1-acyl-GPE, 1-acyl-glycerophosphoethanolamine; 1-acyl-GPC, 1-acyl-glycerophosphocholine; PtdCho, phosphatidylcholine; PtdEth, phosphatidylethanolamine; SM, sphingomyelin. Enzyme abbreviations: CCT, phosphocholine cytidylyltransferase; ChK, choline kinase; CPT, diacylglycerol cholinephosphotransferase; CTL, choline transporter-like protein; EtT, ethanolamine transporter; ECT, phosphoethanolamine cytidylyltransferase; EPT, diacylglycerol ethanolaminephosphotransferase; ETNK, ethanolamine kinase; GPE-PDE, glycerophosphoethanolamine phosphodiesterase; GDPD6, glycerophosphodiester phosphodiesterase domain containing 6; lyso-PL, lysophospholipase; PEMT, phosphatidylethanolamine N-methyltransferase; PLA2, phospholipase A2; PLC, phospholipase C; PLD, phospholipase D.

Metabolic intermediates of PtdCho metabolism were consistently observed to display abnormal levels with magnetic resonance spectroscopy (MRS) (7). An increased total choline (tCho) signal, which consists of signals from phosphocholine (PC), glycerophosphocholine (GPC), and free choline (Cho), and whose chemical structures are shown in Figure 1, has been detected by ^1^H MRS in all cancers tested so far (7). High-resolution ^1^H MRS, which can resolve the signals from the individual components of the tCho signal, confirmed the increase of PC in breast (8–12), prostate (13, 14), ovarian (15), endometrial (16), cervical (15), and brain cancers (17–19). In some cancer cell types such as isolated breast and ovarian cancer cells, a relative decrease of GPC was observed as well, which led to the suggestion to use a higher ratio of PC/GPC as a marker of tumor progression (20, 21). PC was also reported as a useful imaging biomarker of tumor response to several targeted treatments of cancers, enabling ^1^H MRS to be used for monitoring changes in PC as a non-invasive marker of pharmacodynamic treatment response (22–26). A PC decrease and GPC increase was reported after docetaxel treatment in breast cancer cell lines and xenograft models (27).

Magnetic resonance spectroscopy detection of tCho allows for non-invasive and longitudinal monitoring of deep-seated tumors, and it is currently being evaluated as non-invasive biomarker for cancer diagnosis, monitoring of treatment response, and anticancer drug development. In clinical studies, *in vivo* MRS detection of the tCho signal was proposed as a marker of breast cancer malignancy (8, 28–31). The tCho signal has been used to monitor neoadjuvant chemotherapy of breast tumors in patients (32), and decreased tCho was associated with the pathology-detected tumor response to chemotherapy (10, 12, 33). One limitation of using choline-based ^1^H MRS is the difficulty of resolving the signals of PC and GPC and free choline in the *in vivo* ^1^H MRS setting.

**Figure 2:**
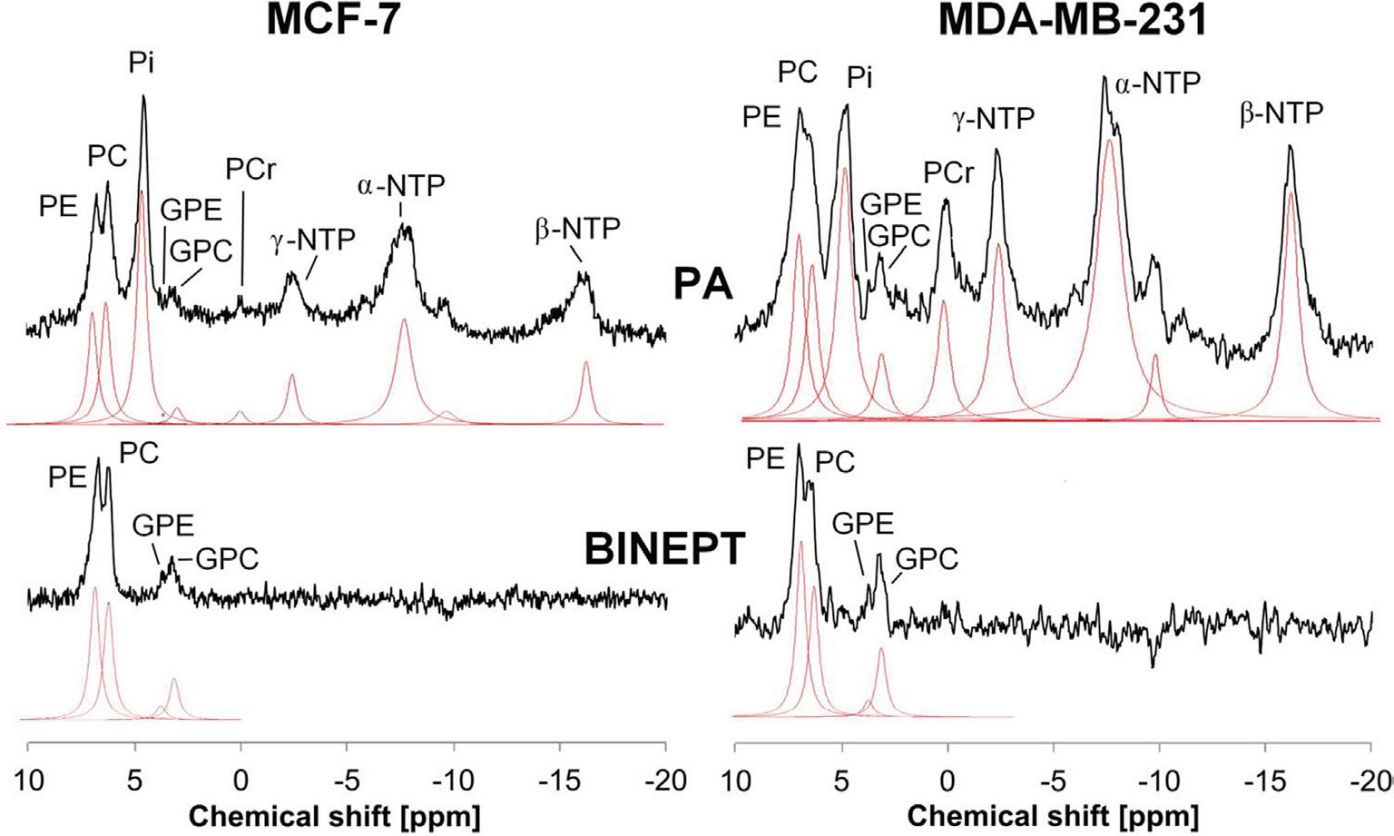
**Example of *in vivo* pulse-acquire (PA, top) and BINEPT (bottom) ^31^P MR spectra of a representative MCF-7 (left) and MDA-MB-231 (right) tumor**. Lorentzian lines as fitted by the software jMRUI (http://www.jmrui.eu/) are shown below each MR spectrum. All phosphorylated metabolites are visible in the PA spectrum, whereas the BINEPT spectrum only contains signals from phospholipid metabolites with H-P-coupling such as phosphoethanolamine (PE), phosphocholine (PC), glycerophosphoethanolamine (GPE), and glycerophosphocholine (GPC). Note the broad, uneven baseline in the 0–5 ppm region of the PA spectra, where signals from mobile membrane phospholipids are resonating. The signal of β-nucleoside triphosphate (NTP) is formed by β-NTP only. The signal labeled α-NTP is an overlapping signal from α-NTP, α-nucleoside diphosphate (α-NDP), nicotinamide adenine diphosphate, and diphosphodiesters. The signal labeled γ-NTP is an overlapping signal from γ-NTP and β-NDP. Typically, β-NTP is the smallest peak of the three NTP signals; however, here, γ-NTP overlaps with a broad baseline signal that makes it appear smaller than β-NTP. Adapted from Wijnen et al. (44).

Phosphorus MRS (^31^P MRS) is also able to detect phospholipid metabolites and displays two sets of phospholipid metabolite peaks: phosphomonoesters (PMEs), which are mainly composed of PC and PE (phosphoethanolamine), and diphosphodiesters, with GPC and GPE (glycerophosphoethonolamine) as the main components as shown in Figure 2. Early ^31^P MRS cancer studies, which were not able to separate the individual components of these two sets of ^31^P peaks very well, detected that PMEs were increased in human breast tumors (34–37), neuroblastomas (38), prostate cancers (39), and hepatic lymphomas (40) as compared to corresponding normal tissues. Following chemotherapy or radiotherapy, a PME reduction was observed in human neuroblastoma (38), breast tumors (35, 36), and hepatic lymphoma (40). Recent developments of innovative ^31^P MRS approaches that are based on ^1^H to ^31^P polarization transfer enable the quantification of *in vivo* levels of PE, PC, GPE, and GPC on small animal and clinical MR scanners (41–44). PE and GPE are the metabolic intermediates of PtdEtn, the second most abundant phospholipid. PE was found to consistently increase in tumors similar to PC (see Figure 2) (45), although its role in cancers is much less explored than PC. The potential of PE and GPE to be monitored is currently being explored along with PC and GPC (41, 44). The changes that occur in tCho, PMEs, and PDEs with oncogenesis and in response to therapy are the result of complex molecular pathways and are therefore not always consistent (8, 37, 46, 47), making it necessary to improve the resolution of clinical *in vivo* MRS applications and to investigate the molecular mechanism underlying these metabolic changes.

Choline uptake and retention can also be imaged using positron emission tomography (PET), mainly with the tracers [^11^C]-choline, [^18^F]-fluoromethylcholine, and [^18^F]-fluoroethylcholine. The use of [^11^C]-choline PET was approved for clinical use in prostate cancer by the federal drug administration of the United States in 2012 (48). Choline PET/computed tomography (CT) is used in the clinic as well and has the advantages of providing improved local disease evaluation and staging of prostate cancer as compared to conventional [^18^F]-FDG PET, giving additional information on nodal staging and suspected metastasis in prostate cancer patients (49).

While choline-based imaging has been explored extensively, discoveries of genes and signaling pathways leading to the changes in choline-containing metabolites are still emerging. The function and regulation of the two enzymes choline kinase α (ChKα) and phospholipase D (PLD) has been more widely explored in cancer, but research into other important enzymes in choline metabolism is still at an early stage. These enzymes may provide new targets for cancer therapy. In this review, we will provide a brief update on the established targets in choline phospholipid metabolism and discuss some new anticancer targets in the choline and ethanolamine phospholipid metabolic pathways as highlighted in red in Figure 1.

## Choline Kinase α

Choline kinase is the enzyme that phosphorylates free choline and produces PC. There are two genes that encode this enzyme: ChKα and choline kinase beta (ChKβ) (50–52). ChKα is well established as an oncogene that promotes tumor initiation and progression (7, 53, 54). Its overexpression and elevated enzyme activity is one of the most established factors that contribute to increased PC and tCho in various tumor tissues including breast (55), ovarian (21), colorectal (56), prostate (56), lung (56, 57), endometrial (16), and pancreatic (58) cancer.

In addition to catalyzing the formation of PC in the Kennedy pathway of PtdCho synthesis, ChKα also has other functions in regulating cell signaling pathways. For example, the non-receptor tyrosine kinase c-Src was shown to phosphorylate ChKα, which in turn forms a protein complex with epidermal growth factor receptor to regulate breast cancer cell proliferation and tumorigenesis (59). Downregulation of ChKα decreased the phosphorylation of the mitogen-activated protein kinase (MAPK) signaling pathway protein ERK1/2 to p-ERK1/2 on T202/Y204 (60), and the PI3K/AKT signaling pathway protein AKT to p-AKT on S473 (61). ChKα inhibition induced exacerbated endoplasmic reticulum (ER) stress and triggered apoptosis *via* the CHOP pathway in cancer cells, but not in the non-tumorigenic mammary epithelial cell line MCF-10A (62). CHOP is the major pro-apoptotic transcription factor that is induced by ER stress. Inhibition of ChKα in cancer cells also resulted in an intracellular increase in ceramides, leading to apoptotic cell death, suggesting that ChKα activity disruption may be a highly specific and selective cytotoxic antitumoral strategy. ChKα knockout mice, but not knockout mice with a loss of ChKβ, are embryonically lethal, implying that ChKα is essential for PtdCho biosynthesis (7, 63, 64).

**Figure 3:**
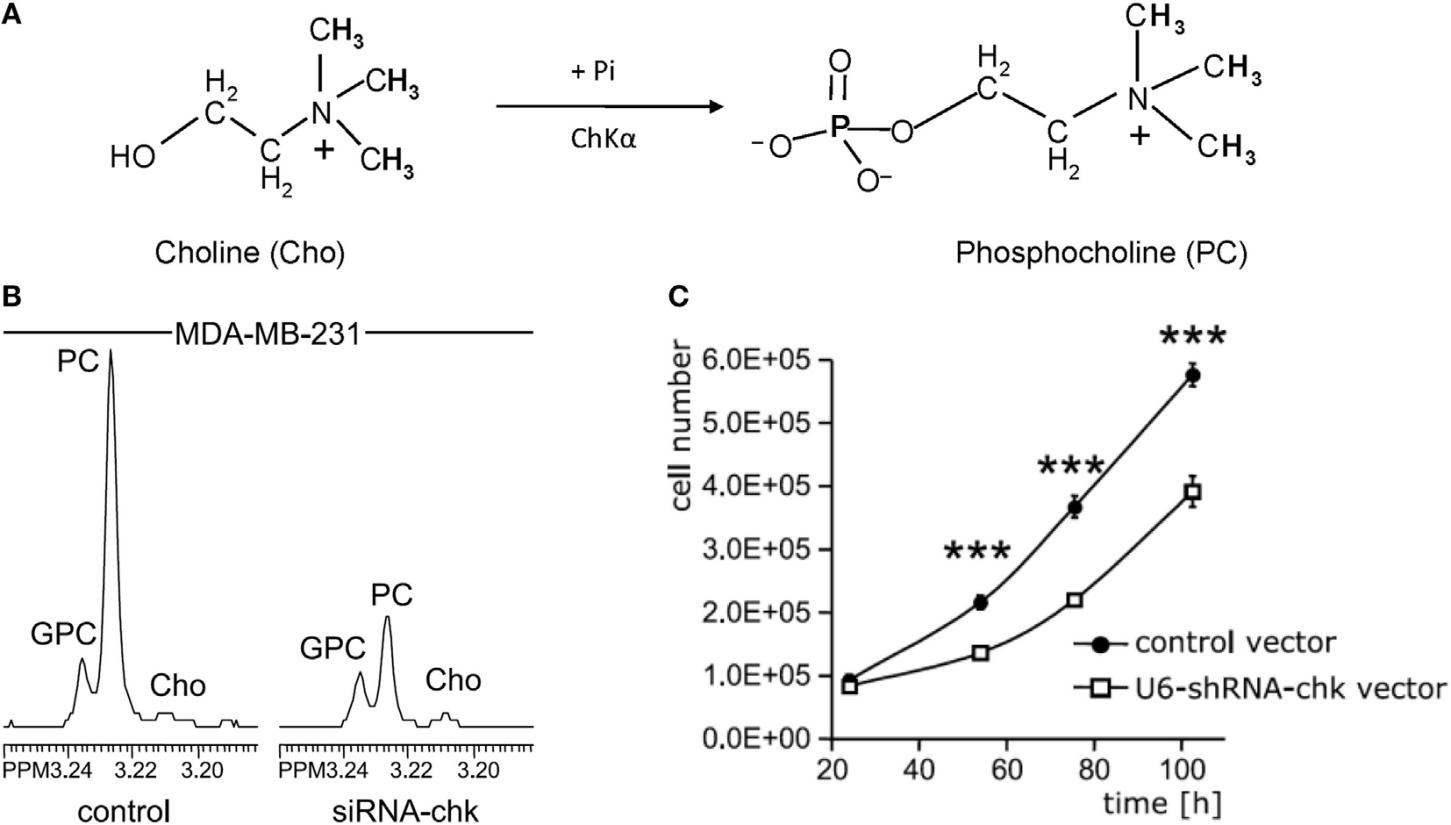
**Choline kinase α (ChKα) as target for anticancer treatment**. **(A)** ChKα converts free choline to phosphocholine (PC). **(B)** Knockdown of ChKα resulted in a dramatic reduction of cellular PC as evident by the decreased PC signal in high-resolution ^1^H MR spectrum obtained from water-soluble metabolites of MDA-MB-231 cells transfected with anti-ChKα siRNA compared to the spectrum from non-target siRNA control. **(C)** Stable knockdown of ChKα with a pSHAG-U6-shRNA vector significantly decreased MDA-MB-231 cell growth rate. Abbreviations: GPC, glycerophosphocholine. ***represents *P* < 0.001, average proliferation rates were calculated from five independent experiments. Adapted from Glunde et al. (69).

Several small molecule inhibitors of ChKα- and siRNA-based nanoparticles have been developed to test their potential for MRS-monitored anticancer treatment. Lacal and colleagues have tested several groups of compounds that inhibit ChKα activity, some of which displayed significant levels of antiproliferative activity and led to a reduction of tumor growth (65–67). One of these inhibitors, TCD-717 (68), is currently being tested in a phase I clinical trial (https://clinicaltrials.gov/ct2/show/NCT01215864). Bhujwalla and colleagues have developed siRNA and shRNA strategies (Figure 3) (69, 70), and siRNA-based nanoparticles against ChKα (71) for anticancer therapy. Both methods of therapy can be monitored by detecting ChKα activity with MRS (22, 69, 70, 72). siRNA silencing of ChKα demonstrated synergistic effects with 5-fluorouracil (5-FU) in the treatment of breast cancer cells (73), and ChKα inhibitors showed similar effects in preclinical studies of treating colorectal cancer (74). Overall, ChKα is a well-established antitumor target, with multiple inhibitors and siRNA-based agents against ChKα under development and in initial clinical testing phases. It is possible that ChKα may enter clinical application as stand-alone treatment or in combination with other first-line chemotherapeutic drugs such as 5-FU. MRS monitoring of tumor tCho or PME levels in patients undergoing treatment with ChKα-targeted drugs may facilitate non-invasive assessment of tumor response for more precise treatment monitoring and dynamic adjustment of the treatment plan.

## PtdCho-Specific Phospholipase D (PLD)

The phospholipase D enzyme hydrolyzes the most abundant phospholipid PtdCho and thereby produces phosphatidic acid (PA) and free choline (75). PA is a second messenger that directly binds to the mammalian target of rapamycin (mTOR) (76). PA can also be converted to other secondary messengers such as diacylglycerol (DAG) by lipid phosphatidate phosphatase, or to lysophosphatidic acid (LPA) by phospholipase A2 (75). There are two isoforms of PLD in the human genome: phospholipase D1 (PLD1) and PLD2, which share 50% amino acid identity and display a similar protein structure (77, 78). PLD1, PLD2, and PA have been reported to interact with several protein partners, forming a molecular network that mediates various cellular functions (79). These cellular functions include vesicle trafficking (endocytosis and exocytosis), trans-Golgi network vesicle formation, cytoskeleton organization, cell migration, cell morphogenesis, autophagy (80), and the regulation of growth, proliferation, survival, and apoptosis (81).

Elevated PtdCho-specific PLD activity and/or increased expression of PLD1 or PLD2 have been observed in many different types of cancer and in transformed cells. A single-nucleotide polymorphism without protein change in PLD1 was reported to increase the risk of small cell lung cancer (82). A polymorphism in PLD2 is associated with the prevalence of colorectal cancer (83). Overexpression of either PLD1 or PLD2 in murine fibroblasts alters their cell growth, confers anchorage-independent growth ability in soft agar, and sarcoma formation in nude mice (84). Genetically enforced overexpression of PLD1 or PLD2 in a human breast cancer xenograft model led to primary tumor initiation and increased metastasis (85). PLD activity was shown to confer rapamycin resistance in human breast cancer cells (86). PLD activity was also demonstrated to couple survival and migration signals in human cancer cells to stress response (87). PLD1 and ChKα are interactive, such that ChKα silencing increases PLD1 expression and PLD1 silencing increases ChKα expression (88). The contributions of PLD to cancer have been summarized by Bruntz et al. as sustaining proliferative signaling, transducing MAPK signaling, regulating mTOR, evading growth suppression, resisting apoptosis- or autophagy-mediated cell death, activating invasion and metastasis, inducing angiogenesis, and deregulating cellular energetics (81).

Targeting PLD for anticancer therapy has been extensively explored. siRNA silencing or genetic ablation of either PLD1 or PLD2 were demonstrated to reduce cell proliferation, cell migration, and/or xenograft growth (85, 89). In ApcMin/+ and azoxymethane/dextran sodium sulfate (AOM/DSS) mouse models, spontaneous and colitis-associated intestinal tumorigenesis was reported to be disrupted by genetic or pharmacological targeting of PLD1, but not PLD2 (90). This was the case because PLD1 inactivation suppressed the self-renewal capacity of colon cancer-initiating cells, which in turn decreased the expression of β-catenin (90). The combination of PLD1 and autophagy inhibition was shown to synergize in inducing tumor cell apoptosis and tumor regression, providing a potential rational target for anticancer therapy (91). In earlier studies, short-chain primary alcohols were used as PLD inhibitor, but concerns were raised about insufficient suppression of PA production and off-target effects (92). Several PLD-specific inhibitors have been screened and evaluated in preclinical studies (81, 92, 93). 5-Fluoro-2-indolyl des-chlorohalopemide (FIPI), a dual inhibitor of PLD1 and PLD2 (93), has shown anticancer potential. Delivering FIPI with an osmotic pump to a xenograft model of breast cancer in mice was able to inhibit primary tumor growth and reduce metastatic growth of nodules in the axillary lymph nodes and lungs (85). Tumors grew more slowly in FIPI-treated mice and showed a significant decrease in microvessel density as compared to non-treated control mice (94). Later studies have explored the anticancer properties of isoform-selective inhibitors of PLD1 or PLD2 (95). The PLD1 selective inhibitor VU0155069 was shown to attenuate intestinal tumorigenesis in the ApcMin/+ and AOM/DSS mouse models (90). Both PLD1 and PLD2 selective inhibitors were found to enhance the radiosensitivity of breast cancer cells (96).

**Figure 4:**
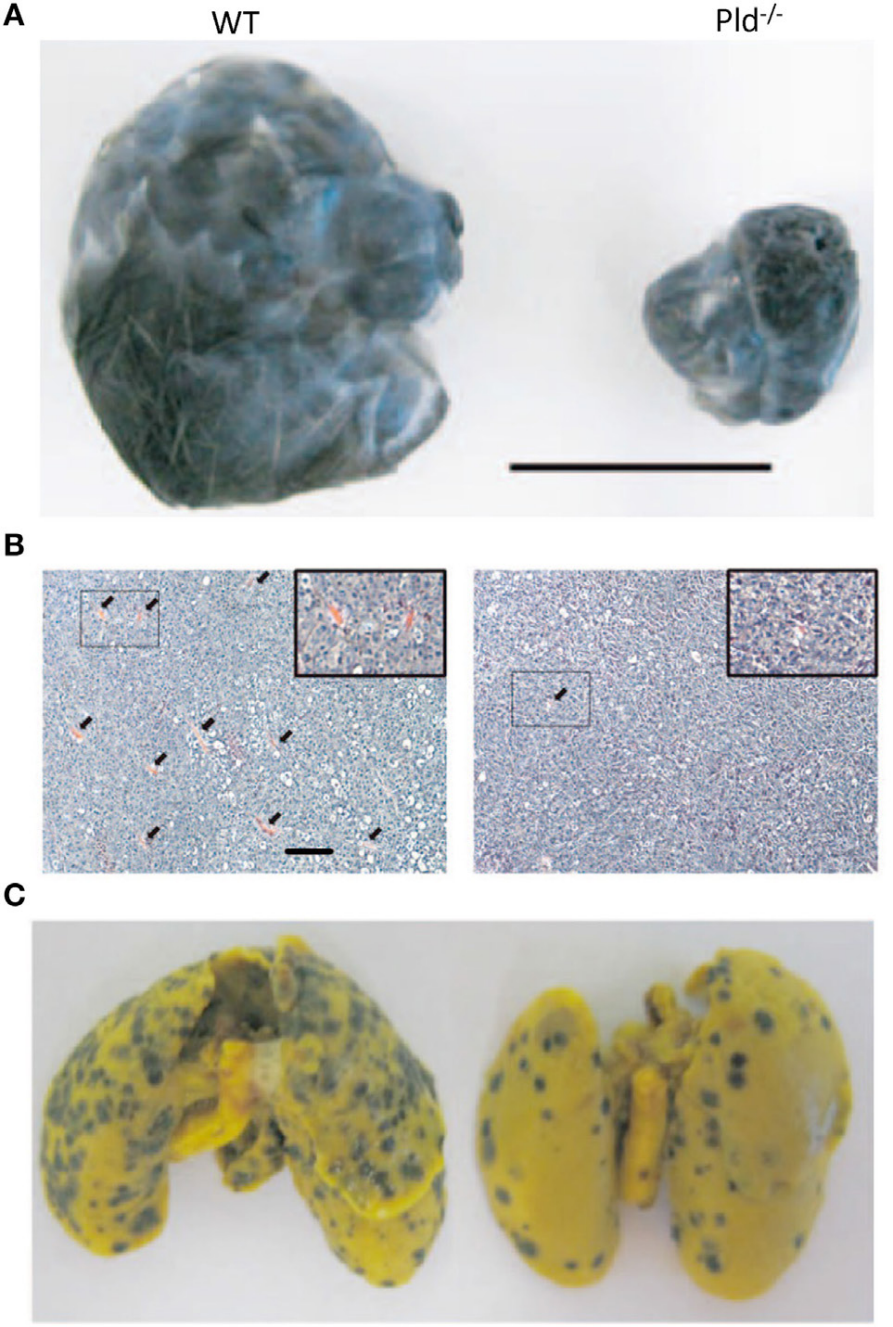
**Phospholipase D1 activity in the tumor microenvironment is important for tumor growth and metastasis**. **(A)** Representative picture showing that melanoma xenografts grown in Pld^−/−^ mice have limited growth volume compared to that growing in wild-type (WT) mice. Scale bar, 1 cm. **(B)** Melanoma xenografts growing in Pld^−/−^ mice have fewer blood vessels as compared to those grown in wild-type mice, as evident from H&E stains of the respective tumor sections. Arrowheads in panel **(B)** indicate blood vessels. Scale bar, 50 µm. **(C)** For melanoma xenografts, lung metastasis has been significantly lower in lungs of Pld^−/−^ mice than in lungs of wild-type mice. Adapted from Chen et al. (94).

The generation of PLD1-deficient transgenic mice provided the opportunity of studying the role of PLD1 in the tumor microenvironment. Tumor xenografts growing in Pld1^−/−^, but not in Pld2^−/−^, mice were demonstrated to have limited primary tumor growth and reduced lung metastasis as shown in Figure 4 (94). Further examination showed that vascular endothelial cells of PLD1-deficient mice displayed reduced activities in signaling pathways that affect vascular endothelial growth factor as a downstream target (94). These signaling pathways were mediated by reduced phosphorylation of Akt, the MAPK proteins ERK1/2, and P38, leading to decreased integrin-dependent cell adhesion to and migration on extracellular matrices, as well as reduced tumor angiogenesis (Figure 4B) (94). Partly mediated by impaired activation of α_IIb_β_3_ integrin in platelets, tumor cell–platelet interactions decreased in Pld1^−/−^ mice, resulting in reduced seeding of tumor cells into the lung parenchyma (Figure 4C) (94). Small molecule inhibitors of PLD1 displayed similar effects on tumors as the genetic ablation of PLD1 in the host mice (94).

Both Pld1^−/−^ and Pld2^−/−^ mice developed overtly normal (80, 97, 98), indicating that PLD1 inhibitors may have low toxicity. Although the existing small molecule inhibitors against PLD1 only impair cell migration and under-perform in killing cancer cells or reducing the proliferation rate of cancer cells as a stand-alone treatment, they may synergize well with other therapy approaches in combatting cancers, due to PLD1’s multiple roles in important cell functions. Moreover, PLD1’s role in the tumor microenvironment, which confers the ability to disrupt tumor angiogenesis in some studies (94), makes it an attractive target. Although no PLD1 inhibitors have entered into clinical trials for anticancer therapy yet, promising results emerging from recent preclinical studies may pave the way for PLD1 inhibitors into clinical studies in the near future.

## Phosphatidylcholine-Specific Phospholipase C (PtdCho-PLC)

Phosphatidylcholine-specific phospholipase C cleaves the PC moiety from PtdCho, thereby generating and releasing DAG and PC into cytosol. DAG is an important second messenger that activates protein kinase C and induces mitogenic signal transduction. Considering the large abundance of PtdCho, it is possible that PtdCho-PLC hydrolysis of phospholipids can produce a more sustained DAG elevation than phosphatidylinositol-specific PLC cleavage of phosphatidylinositol (99). The sustained mitogenic signal produced by PtdCho-PLC generated DAG may lead to cell transformation. Indeed, NIH3T3 fibroblasts stably transfected with bacterial PtdCho-PLC displayed chronically elevated levels of DAG and PC, and a transformed phenotype that was characterized by anchorage-independent growth in soft agar, formation of transformed foci in tissue culture, and loss of contact inhibition (100). Increased PtdCho-PLC enzyme activity was observed in ovarian cancer cells (101, 102), breast cancer cells (103), and squamous cell carcinoma cells (104) as compared to the corresponding non-malignant, immortalized cells. PtdCho-PLC selectively accumulated in raft domains in the plasma membrane of HER2-overexpressing breast cancer cells, in which it colocalized with HER2 (105).

Since the genes encoding PtdCho-PLC have not yet been cloned from mammalian genomes, it is currently not possible to genetically ablate PtdCho-PLC activity, and mechanistic PtdCho-PLC studies have to rely on small molecule inhibitors of PtdCho-PLC, such as tricyclodecan-9-yl-potassium xanthate (D609). D609 competitively inhibits PtdCho-PLC activity and was shown to confer antiviral and antitumor effects (106). Inhibition of PtdCho-PLC with D609 reduced the cellular PC content and blocked cell proliferation in human ovarian cancer cells (102). D609 treatment of human MDA-MB-231 breast cancer cells led to a loss of mesenchymal traits, such as decreased Vimentin and N-Cadherin expression, and a changed cell morphology more similar to mature breast epithelial cells, as well as growth arrest and reduced migration and invasion potential (103). PtdCho-PLC inhibition with D609 also resulted in enhanced HER2 internalization and lysosomal degradation, strong retardation of HER2 re-expression on the membrane, and reduced HER2 content in HER2-overexpressing breast cancer cells, suggesting that PtdCho-PLC inhibitors may be potential candidates for the treatment of HER2-amplified or trastuzumab-resistant breast cancers (105). D609 treatment of the two squamous cell carcinoma cell lines A431 and CaSki, which display different stemness potential, led to reduced cell proliferation, decreased sphere-forming efficiency, and down-modulated mRNA levels of stemness-related markers (104). Although the mammalian PtdCho-PLC genes have not yet been identified, it is evident that PtdCho-PLC activity is important in cell transformation and cancer progression, and that inhibiting PtdCho-PLC activity may have potential as an anticancer strategy. Efforts should focus on cloning mammalian PtdCho-PLC gene(s) and identifying compounds that inhibit PtdCho-PLC and are suitable for clinical application.

## Sphingomyelinases (SMAs)

Sphingomyelin is a sphingosine-based phospholipid that exists in cell membranes with a typical mole percentage of around 10–20 (107). Sphingomyelin typically contains a PC or sometimes a PE headgroup similar to glycerol-based phospholipids. SMAs are enzymes that act analogous to phospholipase C, release PC or PE from sphingomyelin, and thereby generate ceramide (108). According to the pH for their optimum enzyme function, they are classified as acidic, neutral, and alkaline SMAs (109).

The importance of acid sphingomyelinase (ASM) function in tumor irradiation has been well established with genetic studies (110). Niemann–Pick patients have an inherited deficiency of ASM activity, which results in their lymphoblasts failing to respond to ionizing radiation with ceramide generation and apoptosis (110). ASM knockout mice also displayed failure of radiation-induced ceramide generation and apoptosis *in vivo* (110). MCA/129 fibrosarcomas and B16F1 melanomas grown in ASM-deficient mice grow much faster than in control mice and showed reduced endothelial apoptosis upon irradiation (111). When these cells were grown in severe combined immunodeficient mice with ASM-deficiency, the xenografts acquired resistance to single-dose radiotherapy (112), implying that ASM-mediated, radiotherapy-induced apoptosis occurs both in cancer cells and in the tumor microenvironment. Production of ceramide after tumor irradiation or treatment with chemotherapeutic agents, including commonly used daunorubicin, doxorubicin, cisplatin, paclitaxel, gemicitabine, among others, is a well-known treatment response (113). The production of ceramide through hydrolysis of sphingomyelin by ASM following chemotherapy is an important mechanism of cellular ceramide increase which leads to tumor cell apoptosis, as siRNA knockdown of ASM prevents cancer cell death induced by cisplatin (114) or gemcitabine (115, 116).

**Figure 5:**
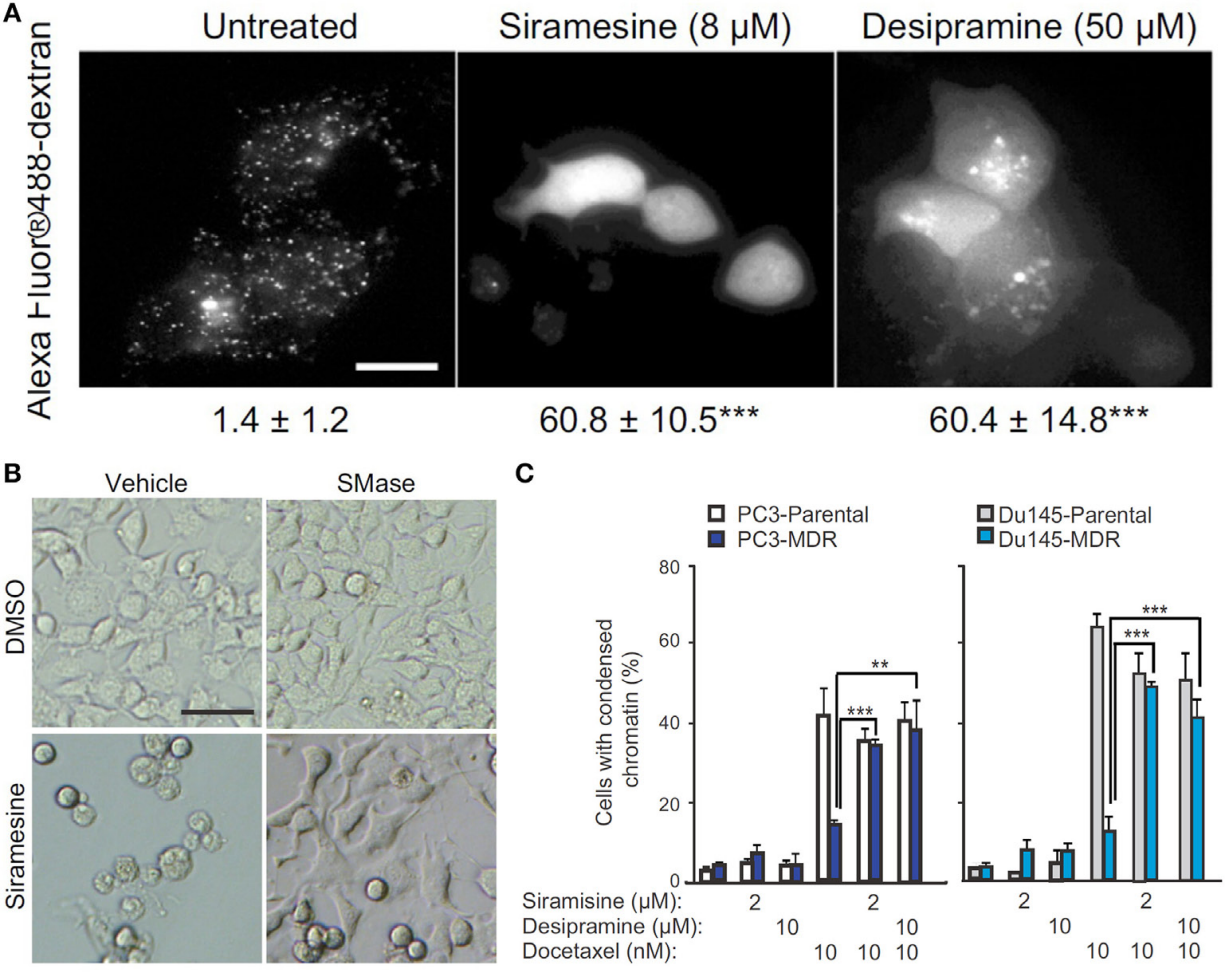
**Targeting acid sphingomyelinases (ASM) is an effective strategy to overcome drug-resistant cancer through lysosome destabilization-mediated cell kill**. **(A)** Siramesine and Desipramine, two cationic amphiphilic drugs and inhibitors of ASM, cause lysosomal membrane permeabilization in MCF-7 cells. The numbers given underneath each image are the respective percentage of cells with lysosomal membrane permeabilization as indicated by cytosolic Alexa Fluor 488-dextran staining. Scale bar, 20 µm. **(B)** Cell death of transformed NIH 3T3-c-src^Y527F^ cells caused by siramesine can be rescued by adding 75 µM ASM from *Bacillus cereus* prior to starting drug treatment. Scale bar, 50 µm. **(C)** ASM inhibition reverts drug resistance of cancer cells. Siramesine and Desipramine treatment of multidrug-resistant (MDR) PC3-MDR or Du145-MDR greatly increased the percentage of cells undergoing apoptotic cell death as evident by condensed chromatin when co-treated with docetaxel. **represents *P* < 0.01, ***represents *P* < 0.001. Adapted from Petersen Nikolaj et al. (121).

The molecular mechanism by which ASM promotes cell apoptosis is mainly through its ceramide-producing ability. Moreover, the formation of ceramide-enriched lipid domains can trap and cluster specific membrane receptors such as CD95 and intracellular signaling molecules, and thus, facilitate cellular signal transduction, which leads to apoptosis, autophagic responses, and cell cycle arrest (114, 117, 118). To improve the therapeutic efficacy of ceramide-producing chemotherapy, further increasing the ceramide generation in cancer cells through activation of SMAs may be a worthwhile strategy to enhance anticancer treatment. However, it is generally hard to activate protein function. On the contrary, most rational drug design strategies inhibit rather than activate protein function. Based on this reasoning, ASM does not seem to be a suitable anticancer target at first sight. However, on the other hand, ASM-deficient human patients suffering from Niemann–Pick disease have cells with lysosomal storage disorders and significantly decreased lysosomal stability (119), suggesting that ASM is essential for maintaining normal lysosomal integrity. To escape from apoptosis, tumor cells, especially therapy resistant cancer cells, probably decrease their ASM activity to a critically low level, making them more sensitive than normal cells to ASM inhibition that can lead to lysosomal destabilization (120). Siramesine and several clinically relevant ASM inhibitors were found to trigger cancer-selective lysosomal cell death, displaying great anticancer potential as evident by reducing tumor growth *in vivo*, and reverting multidrug resistance (Figure 5) (121). So the strength of escaping from apoptosis by decreasing ASM levels becomes a weakness of cancers, through which cancer-specific killing agents can be developed especially for malignant and multidrug-resistant cancers (122).

There are several genes in the mammalian genome encoding neutral sphingomyelinases (N-SMase): the nSMase1 gene most likely encodes a lyso-platelet activating factor with phospholipase C activity (123), the nSMase2 and nSMase3 genes encode *bona fide* SMAs (124), and the newly identified MA-nSMase is a mitochondria-associated protein (125). Similar to ASM, N-SMase activity also promotes apoptosis through production of ceramide (124). The antitumoral chemotherapeutic reagents daunorubicine and arabinoside-C activate N-SMase in leukemic cells, resulting in ceramide release, which activates Jun-N-terminal kinases and Lyn protein tyrosine kinase to mediate cell apoptosis (126–128). Doxorubicin also induces nSMase2 mRNA and protein in a dose-dependent manner, and nSMase2 knockdown can prevent doxorubicin-induced growth arrest (129).

Alkaline sphingomyelinase activity is mostly found in the gastrointestinal tract and in human bile (130), and it is associated with colon cancer. Early studies showed that dietary sphingomyelin intake inhibited the formation of colon cancer that was induced with the carcinogen 1,2-dimethylhydrazine in mice (131). Both, human colorectal carcinoma and familial adenomatous polyposis, display a marked reduction in alkaline sphingomyelinase activity (130, 132, 133). Knockout of the alkaline sphingomyelinase encoding gene NPP7 in mice significantly enhanced colonic tumorigenesis as compared to wild-type mice, after both groups of mice had been treated with a carcinogenic formulation of azoxymethane and dextran sulfate sodium (134). Loss of function mutated NPP7 gene copies were identified in the colon cancer cell line HT-29, the liver cancer cell line HepG2, and in liver tumor tissues (135, 136).

In summary, the family of SMAs generally acts in an anticancer role through ceramide release leading to apoptosis. Radiation therapy, chemotherapeutic agents, and some targeted therapy inhibitors exerted their tumor killing ability by activating sphingomyelinase enzyme activity. The functioning of ASM in lysosomal integration has lead to the concept of inhibiting ASM to destabilize lysosomes and kill cancer cells (120, 122). The cationic amphiphilic drug siramesine, initially developed as an anti-depression agent which failed for having no effect, may be alternatively developed as an anticancer drug, and may be particularly successful when combined with microtubule-destabilizing antimitotic drugs (137). To date, the effects of sphingomyelinase activation or inhibition on the choline metabolite profile and tCho levels have not yet been studied.

## Choline Transporters

The accelerated synthesis of PtdCho to satisfy the need of fast proliferation of cancer cells requires a large quantity of choline as precursor. Cancer cells typically take up free choline from their environment, which in some cases is a rate-limiting step in forming PC and in the Kennedy pathway (138). Free choline, which is an organic cation, cannot cross cell membranes freely, and hence requires active transporters for its import into cells. The human genome has four groups of proteins with choline transport ability: choline transporter 1 (CHT1/SLC5A7), choline transporter-like proteins (CTLs), organic cation transporters (OCTs), and organic cation/carnitine transporters.

A microarray survey of CTL1 revealed that it was expressed in cancers of the central nervous system, ovary, breast, prostate, and leukemia, while highly expressed in melanoma, renal, and colon cancer (139). Functional expression analysis showed that the expression of the CTL family was different across different cancer cell lines, and that cancer cells expressed at least one set of choline transporters to guarantee the import of this essential nutrient. Both the expression of choline transporter genes and the rate of choline uptake in breast cancer cells were shown to be much higher than in the non-malignant human mammary epithelial cell (HMEC) line MCF-10A (140). CHT1 mRNA was only slightly expressed in the neuroblastoma cell lines SH-SY5Y and LA-N-2 (140), while CTL1 was the major choline transporter in these cancer cells (141). CHT1 and OCT2 were upregulated in breast cancer cell lines as compared to HMEC (142), while another study showed that CTL1 and CTL2 were the two genes overexpressed in the breast cancer cell line MCF-7 as compared to the non-malignant mammary epithelial cell line MCF-10A, and CTL2 specifically was detected in human breast cancers by immunohistochemical staining (140). CTL1 was highly overexpressed in human pulmonary adenocarcinoma tissues as compared to matched normal tissues (143). CTL1 was also the major choline transporter that accounts for the enhanced choline uptake in lung adenocarcinoma cell lines, the human HT-29 colon carcinoma cell line (144), leukemia cells (140), and human neuroblastoma cells (141).

The requirement for an increased supply of choline implies that cancer cells may be vulnerable to choline transporter inhibition. Organic cation drugs, which likely share the same transporters with choline, might inhibit choline uptake, and thereby reduce cancer cell viability. The choline analog hemicholinium-3 (HC-3) and tetrahexylammonium chloride were reported to inhibit choline uptake and reduce cell proliferation when treating the human colon carcinoma cell line HT-29 (144). The Na^+^/H^+^ exchanger inhibitor dimethylamiloride and various organic cations such as quinine, quinidine, desipramine, imipramine, clomipramine, diphenhydramine, and fluvoxamine, among others were shown to inhibit choline uptake and cell viability in the small cell lung carcinoma cell line NCI-H69 (145). In the same study, HC-3 and CTL1 siRNA were shown to inhibit choline uptake and cell viability as well, along with increasing caspase-3/7 activity (145). CTL1 inhibitors significantly block choline uptake in the lung adenocarcinoma cell lines A549, H1299, and SPC-A-1, and their ability to block uptake was closely associated with their efficacy in reducing cell proliferation (143). HC-3 also significantly inhibited choline uptake in both HL-60 and Jurkat leukemia cells, thereby leading to increased caspase-3/7 activity and decreased cell viability (140).

**Figure 6:**
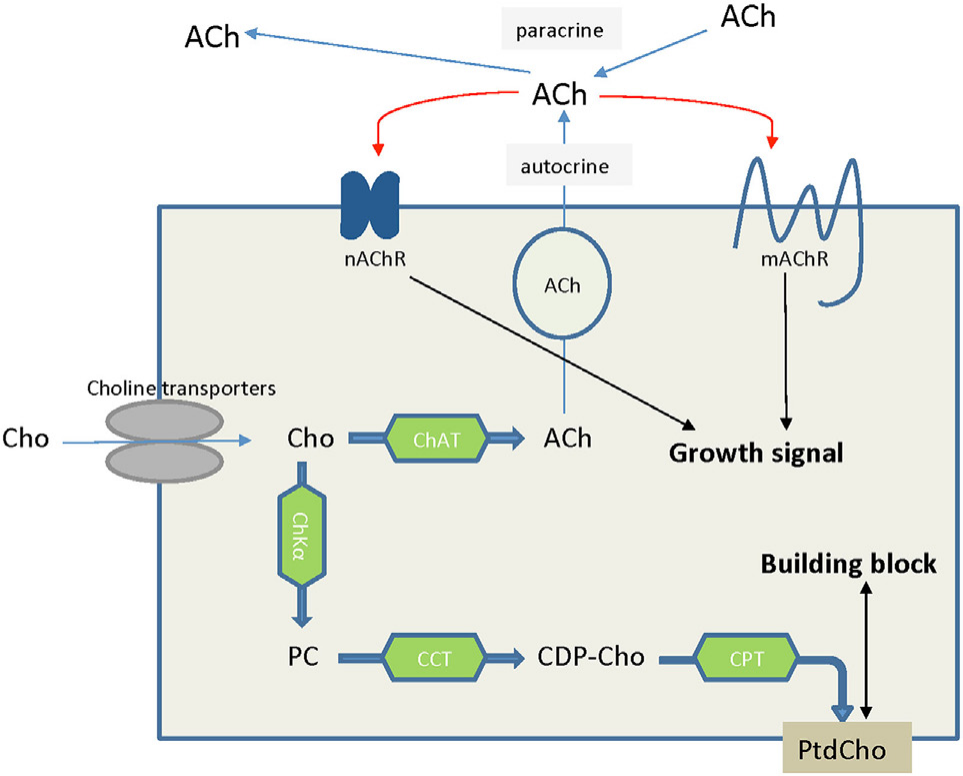
**Choline is an essential nutrient transported into cells by choline transporters**. In some cancer types such as lung cancers, choline that is taken up into cells is converted to acetylcholine, serving as an autocrine or paracrine growth factor which stimulates cancer cell growth. Alternatively, choline is converted to phosphocholine and ultimately phosphatidylcholine, serving as building block to satisfy the increased proliferation rate of cancer cells and tumor growth. Abbreviations: ACh, acetylcholine; CCT, phosphocholine cytidylyltransferase; CDP-Cho, cytidine 5′-diphosphocholine; ChAT, choline acetyltransferase; ChKα, choline kinase α; Cho, choline; CPT, diacylglycerol cholinephosphotransferase; nAChR, nicotinic acetycholine receptor; mAChR, muscarinic acetycholine receptor; PC, phosphocholine; PtdCho, phosphatidylcholine.

The high-affinity choline transporter CHT1 was thought to be unique to cholinergic neurons, in which choline is used to synthesize the neurotransmitter acetylcholine (ACh) (146). However, there is evidence that non-neuronal cholinergic systems exist in lung cancers (147, 148), colon adenocarcinoma (149), and leukemia (140). In these cancers, ACh acts as an autocrine or local paracrine growth factor that stimulates tumor growth, with muscarinic cholinergic receptor subtype M3 or nicotinic cholinergic receptors functioning as its receptors (147, 149, 150), which is indicated in Figure 6. However, the free choline transported into some cells for this non-neuronal ACh synthesis is not dependent on CHT1 transport (150–152). Knockdown of CTL4, but not other genes in the CTL family, in both lung and colon cancer cells significantly decreased ACh secretion and cell growth, suggesting that CTL4 is a reasonable target for certain types of cancer therapy without affecting neuronal ACh synthesis (150, 153). CTL1 was reported to be associated with ACh production in human neuroblastoma cells (141), but negatively associated with ACh secretion from non-small cell lung carcinoma (154). CTL1 inhibition to reduce ACh production may be an effective way of killing cancer cells in certain types of cancer.

Choline transporters are crucial for cells to import extracellular choline. Inhibition of choline transport would likely lead to choline deficiency and ceramide accumulation, which in turn would trigger apoptosis *via* p53-independent pathway (140, 155, 156). Overall, choline transporters, and in particular CTL1 and CTL4, are interesting targets for anticancer drug development because their inhibition has a great potential to reduce proliferation and induce apoptosis or block the tumor promoting ACh-M3 muscarinic cholinergic receptor system. As CHT1 is necessary for neuronal ACh biogenesis, as evident by the lethal effect of CHT1 knockout in mice (157) and the severe toxicity of its inhibitor hemicholinium-3 (158), developing inhibitors specific to CTLs with low affinity to CHT1 is a must in targeted drug development to avoid possible toxic side effects.

## Glycerophosphodiesterases

**Figure 7:**
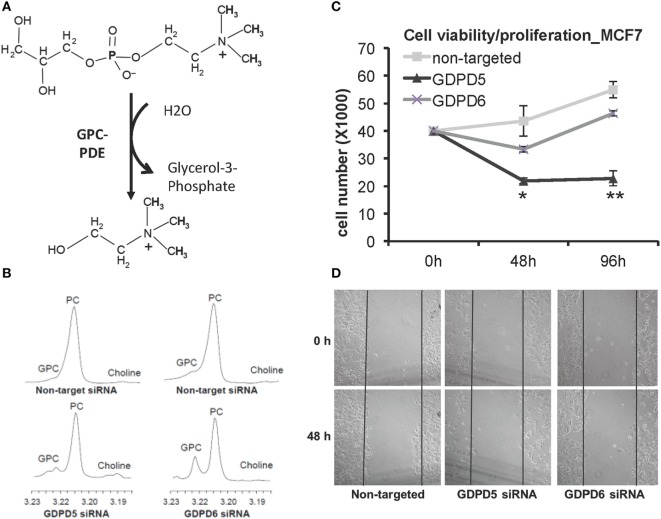
**GDPD5 and GDPD6, the two glycerophosphocholine phosphodiesterases (GPC-PDEs) reported to release choline from glycerophosphocholine (GPC), show potential anticancer effects**. **(A)** GPC-PDE enzyme activity is defined as cleaving the choline moiety from GPC and thereby generating glycerol-3-phosphate. **(B)** Transient knockdown of GDPD5 or GDPD6 by siRNA treatment of MDA-MB-231 cells shows that GDPD6 silencing leads to a significant elevation of the GPC peak in high-resolution ^1^H MR spectra from water-soluble metabolites, while GDPD5 silencing marginally increases GPC levels in this cell line. **(C)** GDPD5, but not GDPD6, siRNA shows cytotoxic effect in MCF-7 breast cancer cells indicated by reduced cell numbers following siRNA treatment of MCF-7 cells. **(D)** Both GDPD5 and GDPD6 silencing decreased MCF-7 cell migration detected by scratch assay. Abbreviations: PC, phosphocholine. *represents *P* < 0.05, **represents *P* < 0.01. Adapted from Cao et al. (163).

Glycerophosphodiester phosphodiesterases (GDPDs) or glycerophosphodiesterases (GDEs) are enzymes that use glycerophosphodiesters as substrates and break them down into glycerol 3-phosphate and alcohols (see Figure 7A). Although bacterial GDPDs can hydrolyze various glycerophosphodiesters, the mammalian genes display substrate preference, and the substrates are not limited to glycerophosphodiesters (159). The human genome encodes seven members of the GDE family, and three of them, as well as their close homologs in mice, were reported to hydrolyze glycerophosphodiesters (159). GDE1 displays substrate preference toward glycerophosphoinositol (GPI), while glycerophosphoserine (GPS) and glycerophosphoglycerate (GPG) are direct GDE1 substrates as well, and GPE is able to block the glycerophosphoinositol phosphodiesterase activity of GDE1, but not GPC (160). The two GDPDs: GDPD5 (or GDE2) and GDPD6 (or GDE5, GPCPD1) were reported to display glycerophosphocholine phosphodiesterase (GPC-PDE) activity (161, 162).

GDPD5 was first reported in mice as a GPC-PDE that osmotically regulates GPC levels in renal medullary cells, in which GPC is an abundant and important osmoprotective organic osmolyte (161). Phosphorus MRS measurements of MDA-MB-231 breast cancer xenografts with constitutively silenced GDPD5 in nude mice also displayed an increase in GPC (164). GDPD5 expression was found to correlate with breast cancer malignancy (165). GDPD5, as well as PLD1 and ChKα, were highly expressed in estrogen receptor negative (ER^−^) breast cancers, which also displayed higher PC, tCho, and lower GPC in comparison with ER+ cases (165). High-resolution ^1^H MRS analysis of breast cancer cell lines in which GDPD5 or GDPD6 were silenced demonstrated that GDPD6 has a leading role in regulating the intracellular GPC level in breast cancer cells (Figure 7B) (163). siRNA silencing of GDPD5 reduced the viability (Figure 7C) and migration (Figure 7D) of ER + MCF-7 breast cancer cells, and decreased the migration and invasion of triple-negative MDA-MB-231 breast cancer cells (163). GDPD5 also has important functions in regulating neuron differentiation through glycosylphosphatidylinositol-anchor cleavage of reversion-inducing cysteine-rich protein with kazal motifs (RECK) (166), which, further downstream, leads to inactivation of the Notch signaling pathway.

GDPD6 was shown to be an Mg^2+^-dependent GDE that is able to hydrolyze GPC and GPE, but does not show activity with GPG, GPI, and GPS as substrates (167). The role of GDPD6 in cancer was recently identified, demonstrating that GDPD6 expression promotes cancer cell migration and invasion through protein kinase alpha signaling (162). GDPD6 mRNA levels were shown to be increased in metastasizing as compared to non-metastasizing endometrial carcinomas, and GDPD6 expression was negatively associated with relapse-free survival in endometrial and ovarian cancers (162). GDPD6 was overexpressed in endometrial cancers, along with ChKα overexpression, resulting in a 70% increase in PC levels (16). Microarray analysis of GDPD6-silenced MCF-7 and OVCAR-3 cells compared to control samples revealed that the expression of integrin β1 was reduced, which resulted in decreased cell attachment and spreading, while overexpression of GDPD6 had the opposite effect (168). The GDPD6 protein contains two conserved domains, which are a carbohydrate-binding domain and a catalytic domain conferring GDE enzyme activity. GDPD6 was also shown to regulate skeletal muscle development in an enzyme activity-independent domain (167). It is not yet clear whether the choline-releasing enzymatic activity of GDPD6 is important for its function in promoting cancer cell migration and invasion.

Both GDPD5 and GDPD6 are emerging as potential anticancer targets in choline metabolism. Their differing specific roles in conferring cancer aggressiveness and degrading GPC are at a relatively early stage of investigation and will require additional mechanistic and preclinical studies to evaluate their full potential as possible drug targets for anticancer therapy.

## Phosphatidylethanolamine N-Methyltransferase (PEMT)

Phosphatidylethanolamine can be directly converted to PtdCho in one step through three methylations of the ethanolamine moiety of PtdEtn, which is catalyzed by the enzyme PEMT. This enzyme was initially isolated from rat liver and was thought to be specific to the liver (169). The first indication that PEMT can suppress cancer was the discovery that the growth of rat hepatoma cells was suppressed by overexpressing the PEMT gene (170). PEMT is highly expressed in normal liver, but its activity was negligible in the two hepatoma cell lines and almost diminished during chemically induced hepatocarcinogenesis (171, 172), and the induced neoplastic phenotype could be partially reversed by PEMT cDNA transfection (172). When growing hepatoma cells in an animal host, PEMT activity and protein expression were barely detected in the fast proliferation stage, while its mRNA and protein reappeared in stationary growth stages (173). Forced expression of PEMT in McArdle-RH7777 hepatoma cells resulted in cell apoptosis (173).

Most early studies of PEMT function were using non-human hepatoma cells or chemically induced hepatocarcinogenesis models in animals. A later study with clinical samples of human hepatocellular carcinomas (HCC) showed that while the PEMT gene was intact, its mRNA level was reduced or even absent compared to peritumoral normal tissue, which was also observed in parallel for PEMT’s enzyme activity (174). PEMT mRNA was found to inversely correlate with HCC histological stage, and the absence of PEMT mRNA in tumor tissue from cancer patients was associated with poor survival (174). While early studies of PEMT’s role in cancer were mostly observed in liver cancers, more recent studies indicated that PEMT polymorphism is also related to breast cancer risk (175). In BRCA1-mutated breast cancer, the PEMT gene undergoes epigenetic repression, and hypermethylation of a specific site in the PEMT promoter is associated with histological grade and estrogen receptor status (176). This finding is consistent with the observation that PtdEtn increased with breast tumor grade (6). Another study in small cell lung cancer indicated that an increase in PEMT expression predicted shorter patient survival time, which is opposite to the observations made in liver and breast cancer (177).

The molecular mechanisms by which PEMT expression suppresses hepatoma growth are not yet fully understood. One study showed that PEMT activity negatively correlated with PtdCho synthesis in the Kennedy pathway (178). When PEMT was overexpressed in McArdle-RH7777 rat hepatoma cells, cellular PtdCho levels did not change even though [methyl-^3^H]methionine and [^3^H]ethanolamine were incorporated into PtdCho, suggesting that conversion of PtdEtn to PtdCho occurred (178). Molecular analysis in this study showed that CTP:phosphocholine cytidylyltransferase (CT) activity, which is the rate-limiting step in the Kennedy pathway of PtdCho synthesis, decreased (178). This was most likely the reason for the unchanged PtdCho levels during PEMT overexpression in this study (178). The slowing down of the Kennedy pathway while PEMT activity increased may have contributed to the reduced cell proliferation rate in this study (178). Chemically induced hepatocarcinogenesis and growth of hepatoma cells in host animals also demonstrated opposing activities of PEMT and CT (171, 173). The overexpression of PEMT in CBRH-7919 rat hepatoma cells resulted in a downmodulation of the PI3K/Akt signaling pathway, which was evident by reduced protein levels of c-Met, PDGF receptor, PI3K, Akt, and Bcl-2, in addition to decreased phosphorylation of Akt Thr308, which was accompanied by elevated cell apoptosis (179).

In summary, PEMT, an enzyme that directly converts PtdEtn to PtdCho by sequential methylation, acts as an onco-suppressor. Its function and molecular mechanism of tumor suppression, and its regulation during carcinogenesis are currently starting to emerge and will require more in depth studies.

## Ethanolamine Kinase (ETNK)

The first step in the Kenney pathway toward *de novo* synthesis of PtdEth or PtdCho is the ATP-dependent phosphorylation of free intracellular ethanolamine or free intracellular choline. While ChKα does confer dual choline and ETNK activity with a preference for choline (180), the conversion of free ethanolamine to PE is mainly achieved through ETNK enzyme activity (181, 182). There are two genes encoding ETNK enzymes in the human genome: ETNK1 (or EKI1) and ETNK2 (or EKI2). Forced overexpression of the ETNK1 gene in COS7 cells accelerated the Kennedy pathway of PtdEtn synthesis, but did not result in an accumulation of PtdEtns due to concomitant faster degradation of PtdEtns (183). Interestingly, an ETNK1 recurrent missense mutation was frequently found in systemic mastocytosis with eosinophilia and chronic myelomonocytic leukemia (184, 185). These mutations caused an amino acid change located in the kinase domain, which was predicted to disrupt the enzyme’s catalytic activity (184, 185). Metabolite measurements revealed an averaged 5.2-fold decrease in the PtdEtn/PtdCho ratio of ETNK1-mutated atypical chronic myelomonocytic leukemia samples as compared to ETNK1 wild-type samples (184). In contrast to these findings in leukemia, in different types of human testicular cancers, EKI1 co-amplified with DAD-R and SOX5, all of which reside in close-by chromosomal regions of the short arm of chromosome 12 (12P) (186). The DAD-R gene is thought to be clinically relevant in the malignant transformation of testicular cancers because of elevated DAD-R expression in testicular cancers with 12P amplification, which correlated with invasive growth and a reduced level of apoptosis, as compared to testicular cancers without 12P amplification (186).

While the roles and functions of ETNKs in cancer are still at an early stage of investigation, the product of its enzymatic activity, PE, has been known to be elevated in several cancers for decades (45, 187). Moreover, synthetic PE has recently been suggested to have anticancer effects on melanoma (188), Ehrlich ascites tumor (189), and leukemia (190). PE has also been reported to possess anti-angiogenic and anti-metastatic activity in lung cancer (191) and to induce cell cycle arrest and apoptosis in MCF-7 breast cancer cells (192). While the therapeutic effects of PE still need to be verified by additional studies and eventually clinical trials, ETNK may be an additional target for MRS-monitored anticancer treatment and should be the focus of more research studies in the near future. It is also possible that elevated PE levels in several types of cancer could be the result of elevated ChKα expression and activity levels, which is known to also act on free ethanolamine (180). Additional studies are necessary to clarify the involvement of ChKα in the elevated tumoral PE levels.

## The Future: Targeting Multiple Targets and Metabolic Nodes

As our molecular understanding of the different roles of choline and ethanolamine phospholipid metabolism enzymes in cancer is growing, it will become important to study the interactions between these enzymes as well. For example, in aggressive ER^−^ breast cancers, GDPD5, PLD1, and ChKα, were simultaneously highly expressed, leading to elevated PC and tCho levels (165). Moreover, it was recently shown that by silencing ChKα in breast cancer cells, PtdCho–PLD1 was in turn upregulated and *vice versa* (88). Only silencing of both enzymes simultaneously increased apoptosis in the tested breast cancer cells, supporting the necessity for targeting multiple enzymes in choline phospholipid metabolism as a strategy of choice (88). This is also important as targeting only one choline metabolic enzyme for anticancer treatment may lead to the development of resistance in the treated cancer cells more easily as cancer cells are able to adapt to new growth environments and thereby acquire resistance.

Once the body of knowledge about the regulation of choline and ethanolamine phospholipid enzymes has grown even further, it may be worthwhile pursuing systems biology approaches of network analyses to identify the important nodes in these metabolic pathways that are the most vulnerable for targeting as anticancer treatment strategy. Such types of analyses have already been performed for drug design and development (193) and recently for tissue-specific metabolic networks, including tumors (194).

## Conclusion

The aberrant choline and ethanolamine phospholipid metabolism in cancer has recently further been established and solidified as a universal metabolic hallmark of cancer. However, our current knowledge of druggable targets in choline metabolism is still emerging, with only a few enzymes such as ChKα and PLD1 having been explored to a level where a somewhat complete picture of their regulation, oncogenic roles, and interaction networks in cancer are becoming available. For all other enzymes discussed in this review, a lot more basic and translational research is required to evaluate the exact molecular roles of PtdCho-PLC, SMases, choline transporters, GDEs, PEMT, and ETKN in cancer. Metabolic imaging approaches such as MRS and PET are valuable tools to help assess the targeting and regulation outcomes in these studies and to serve later on as monitoring tools in the clinic to assess the response to treatment. Future studies and computational modeling approaches to evaluate the interaction between choline and ethanolamine metabolic enzymes in cancer will be necessary.

## Author Contributions

MC drafted the manuscript and the figures. KG conceived of the manuscript and guided MC through writing it and edited the final manuscript and figures. ZB edited the manuscript.

## Conflict of Interest Statement

The authors declare that the research was conducted in the absence of any commercial or financial relationships that could be construed as a potential conflict of interest.
